# Comparative Neuropharmacology and Pharmacokinetics of Methamphetamine and Its Thiophene Analog Methiopropamine in Rodents

**DOI:** 10.3390/ijms222112002

**Published:** 2021-11-05

**Authors:** Silja Skogstad Tuv, Marianne Skov-Skov Bergh, Jannike Mørch Andersen, Synne Steinsland, Vigdis Vindenes, Michael H. Baumann, Marilyn A. Huestis, Inger Lise Bogen

**Affiliations:** 1 Department of Forensic Sciences, Oslo University Hospital, 0456 Oslo, Norway; Silja.Skogstad.Tuv@ous-hf.no (S.S.T.); Marianne.Skov-Skov.Bergh@ous-hf.no (M.S.-S.B.); Jannike.Morch.Andersen@ous-hf.no (J.M.A.); Synne.Steinsland@ous-hf.no (S.S.); Vigdis.Vindenes@ous-hf.no (V.V.); 2 Department of Pharmacology, Oslo University Hospital, 0372 Oslo, Norway; 3 Designer Drug Research Unit, Intramural Research Program, National Institute on Drug Abuse, Baltimore, MD 21224, USA; mbaumann@mail.nih.gov; 4 Institute of Emerging Health Professions, Thomas Jefferson University, Philadelphia, PA 19107, USA; marilyn.huestis@gmail.com

**Keywords:** locomotor activity, methamphetamine, methiopropamine, mouse, neuropharmacology, novel psychoactive substance, NPS, pharmacokinetics, pharmacology, psychostimulant

## Abstract

Methiopropamine is a novel psychoactive substance (NPS) that is associated with several cases of clinical toxicity, yet little information is available regarding its neuropharmacological properties. Here, we employed in vitro and in vivo methods to compare the pharmacokinetics and neurobiological effects of methiopropamine and its structural analog methamphetamine. Methiopropamine was rapidly distributed to the blood and brain after injection in C57BL/6 mice, with a pharmacokinetic profile similar to that of methamphetamine. Methiopropamine induced psychomotor activity, but higher doses were needed (E_max_ 12.5 mg/kg; i.p.) compared to methamphetamine (E_max_ 3.75 mg/kg; i.p.). A steep increase in locomotor activity was seen after a modest increase in the methiopropamine dose from 10 to 12.5 mg/kg, suggesting that a small increase in dosage may engender unexpectedly strong effects and heighten the risk of unintended overdose in NPS users. In vitro studies revealed that methiopropamine mediates its effects through inhibition of norepinephrine and dopamine uptake into presynaptic nerve terminals (IC_50_ = 0.47 and 0.74 µM, respectively), while the plasmalemmal serotonin uptake and vesicular uptake are affected only at high concentrations (IC_50_ > 25 µM). In summary, methiopropamine closely resembles methamphetamine with regard to its pharmacokinetics, pharmacodynamic effects and mechanism of action, with a potency that is approximately five times lower than that of methamphetamine.

## 1. Introduction

**Figure 1:**
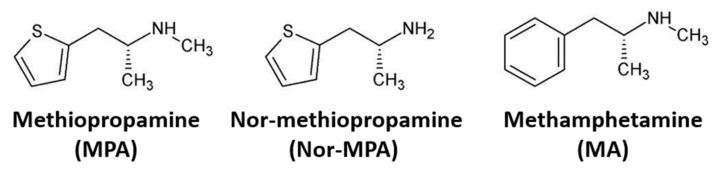
Structural formulas of methiopropamine (MPA), nor-methiopropamine (nor-MPA) and methamphetamine (MA).

The novel psychoactive substance (NPS) methiopropamine (1-(thiophen-2-yl)-2-methylaminopropane) emerged on the European drug market in 2011. Methiopropamine is a structural analog of methamphetamine where the benzene ring of methamphetamine is replaced with a thiophene ring (Figure 1). Methiopropamine is mainly sold as a pure substance, but is also found in combination with other drugs, e.g., the combination of methiopropamine and 2-aminoindane, branded as legal cocaine (“Synthacaine”) [1]. Several web-based vendors offer methiopropamine for online sale, making the substance easy to obtain [2]. According to users, methiopropamine produces effects similar to those of amphetamine, including mental stimulation, alertness and increased energy and focus, with common routes of administration being oral, intranasal and inhalation [3]. Methiopropamine was identified as one of the three most common NPSs in the UK in 2014/2015 [4], while a Norwegian study reported a snapshot prevalence of 0.8% in a population of drivers suspected of being under the influence of drugs in the same time period [5].

Despite its presence as an NPS for several years, little information is available about methiopropamine’s pharmacokinetics and precise mechanism of action. An in vitro study examining the neurochemical profiles of different NPSs reported that methiopropamine is a potent inhibitor of dopamine (DA) and norepinephrine (NE) uptake at human transporters expressed in transfected cells [6]. A pharmacokinetic study analyzing rat and human urine after methiopropamine intake reported the unchanged drug as the main excretion product. In the human urine sample, the *N-*demethylated metabolite nor-methiopropamine and the parent compound were detected 18 h after self-reported intake of 200 mg methiopropamine [7]. Tyrkkö et al. [8] found similar results in human cases where methiopropamine and nor-methiopropamine were the only substances found in urine samples. A case study of acute toxicity after intake of methiopropamine also confirmed the presence of hydroxy nor-methiopropamine in urine, in addition to methiopropamine and nor-methiopropamine, 21 h after nasal insufflation of 50 mg of a powder marketed as “Quicksilver” [9].

Several clinical case reports describe acute toxicity after recreational methiopropamine use. One case with analytical confirmation of methiopropamine described tachycardia, chest pain, anxiety, nausea, vomiting and visual hallucinations as major intoxication symptoms [9], while another presented with agitation, confusion, paranoid delusions, hallucinations and incoherent speech [10]. Several fatal methiopropamine cases were reported, however, in most cases, methiopropamine was found in combination with other substances [11,12,13]. In England and Wales, methiopropamine use was reported as the cause of death in 6 and 23 cases of single and polydrug use, respectively, between 2012 and 2016 [12]. In one fatal case of isolated methiopropamine use, a peripheral postmortem blood concentration of 38 µg/mL was reported [14].

The aim of the present study was to characterize the neuropharmacological effects and pharmacokinetics of methiopropamine, compared with its structural analog methamphetamine. We examined the drugs’ locomotor stimulating effects in mice and drug concentrations in the blood and brain, as well as changes in the post-mortem tissue concentrations of monoamines and their metabolites in brain areas important for reward. Finally, we examined methiopropamine’s mechanism of action by carrying out in vitro studies of drug interactions with monoamine transporter proteins in rodent brain tissue.

## 2. Results

### 2.1. Locomotor Activity Studies

**Figure 2:**
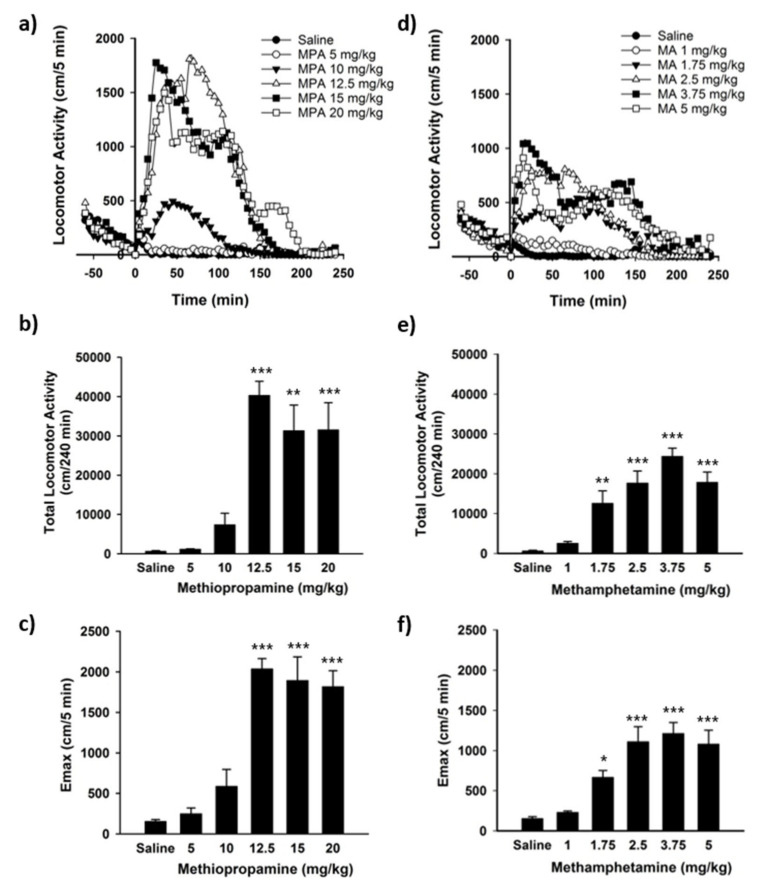
Locomotor activity in mice after injection of (**a**–**c**) methiopropamine (MPA) and (**d**–**f**) methamphetamine (MA). Locomotor activity was recorded for 240 min after i.p. injection of saline (0.9%), MPA (5–20 mg/kg) or MA (1–5 mg/kg). The results are expressed as (**a**,**d**) activity profiles of the mean distance travelled per 5 min intervals, (**b**,**e**) total run distance during the 240 min session and (**c**,**f**) maximum run distance (E_max_) per 5 min intervals, *n* = 4–10. * *p* ≤ 0.05; ** *p* ≤ 0.01; *** *p* ≤ 0.001 vs. saline (Dunnett’s post hoc test).

Injection of methiopropamine resulted in stimulation of locomotor activity (F(5, 24) = 19.20, *p* < 0.001), with significantly increased activity after administration of 12.5–20 mg/kg. Hyperlocomotion was induced immediately following drug injection and declined to zero after 2.5 to 3.5 h (Figure 2a). The total distance travelled and peak effect (E_max_) during the 4 h locomotor session were affected by the dose (total distance (F(5, 25) = 13.47, *p* < 0.001), E_max_ (F(5, 38) = 43.45; *p* < 0.001)), with a maximum total locomotor activity of 40,313 ± 3575 cm (Figure 2b, *p* < 0.001) and peak effect of 2037 ± 127 cm/5 min (Figure 2c, *p* < 0.001) after injection of 12.5 mg/kg. The locomotor activity reached a “plateau effect” at 12.5 mg/kg, with no further increases in the total run distance or E_max_ following administration of higher 15 and 20 mg/kg doses (Figure 2b,c).

Methamphetamine administration also induced hyperlocomotion in mice (F(5, 24 = 20.88, *p* < 0.001) with significantly increased activity after administration of 1.75–5 mg/kg (Figure 2e). As seen for methiopropamine, the locomotor activation appeared immediately after drug administration and declined to zero after 2.5 to 3.5 h (Figure 2d). The total distance travelled and E_max_ during the 4 h locomotor session were affected by the dose (total distance (F(5, 24) = 20.65, *p* < 0.001), E_max_ (F(5, 44) = 24.34; *p* < 0.001)), with a maximum total locomotor activity of 24,401 ± 2043 cm and E_max_ of 1312 ± 126 cm 5 min after administration of 3.75 mg/kg methamphetamine (Figure 2e,f). Increasing the methamphetamine dose to 5 mg/kg did not increase the total run distance or E_max_ compared with the 3.75 mg/kg dose. Injection of saline did not induce changes in locomotor activity in the mice (Figure 2a,d).

### 2.2. Pharmacokinetic Studies

**Figure 3:**
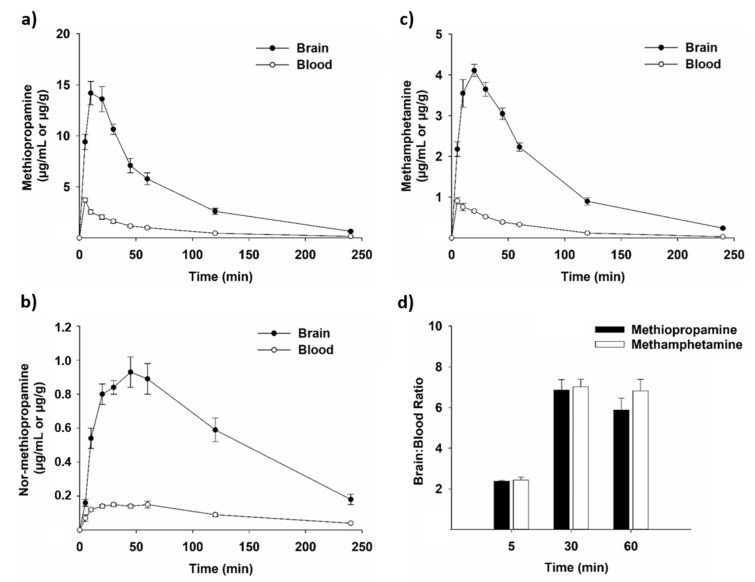
Concentrations of (**a**) methiopropamine, (**b**) nor-methiopropamine and (**c**) methamphetamine in mouse blood (white dots) and brain tissue (black dots) 5, 10, 20, 30, 45, 60, 120 and 240 min after a single i.p. injection of 12.5 mg/kg methiopropamine (**a**,**b**) or 3.75 mg/kg methamphetamine (**c**). Each time point represents mean ± SEM of blood (µg/mL) or brain (µg/g) concentrations, *n* = 4–8. (**d**) The concentration ratio in brain vs. blood for methiopropamine and methamphetamine 5, 30 and 60 min after drug injection, *n* = 5–6.

Doses for the pharmacokinetic studies were chosen based on the doses that induced the highest level of locomotor activity in behavioral assessments (Figure 2). The blood and brain concentrations of methiopropamine and nor-methiopropamine after i.p. administration of 12.5 mg/kg methiopropamine are shown in Figure 3a,b. Methiopropamine was well absorbed into the blood after i.p. injection with a maximum concentration (C_max_) of 3.71 ± 0.21 µg/mL at the earliest time point measured (5 min; Figure 3a). Methiopropamine was rapidly eliminated from the blood, and the concentration was reduced to 12% of C_max_ within 120 min after administration. Methiopropamine was found at high concentrations in the brain tissue, with a C_max_ of 14.19 ± 1.14 µg/g measured 10 min after drug injection (Figure 3a). The elimination half-lives of methiopropamine were estimated to be approximately 31 and 35 min in the blood and brain, respectively.

The metabolite nor-methiopropamine was present at lower concentrations displaying a C_max_ of 0.15 ± 0.01 µg/mL in the blood 30 min after methiopropamine injection, and a C_max_ of 0.93 ± 0.09 µg/g in the brain 45 min after drug injection (Figure 3b). Nor-methiopropamine was still detectable in the blood and brain tissue at the latest time point measured, 4 h after methiopropamine injection.

Methamphetamine reached C_max_ in the blood and brain shortly after i.p. administration (Figure 3c). The C_max_ was 0.91 ± 0.08 µg/mL in the blood at the earliest time point measured (5 min) and 4.11 ± 0.15 µg/g in the brain 20 min after drug injection. The elimination half-lives of methamphetamine in the blood and brain were calculated to be approximately 36 and 45 min, respectively. The concentration vs. time curve profiles of methiopropamine and methamphetamine were almost identical (Figure 3a,c), and no significant differences in the brain:blood ratios were found for the two drugs (*p* > 0.05).

### 2.3. Drug Effects on Neurotransmitter Uptake

**Figure 4:**
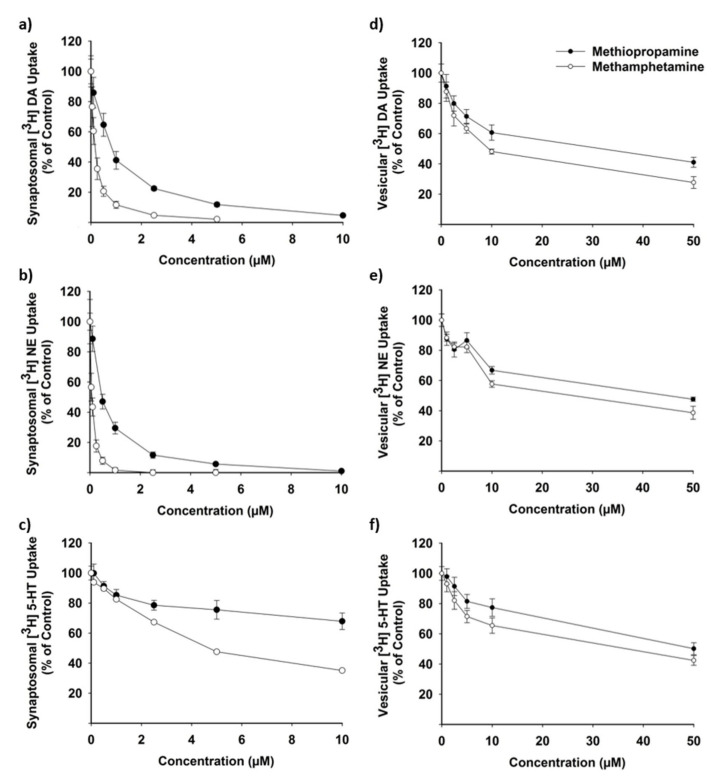
Effects of methiopropamine (0.1–50 µM) and methamphetamine (0.05–50 µM) on (**a**–**c**) synaptosomal and (**d**–**f**) vesicular uptake of [^3^H] dopamine (DA), [^3^H] norepinephrine (NE) and [^3^H] serotonin (5-HT) in rat brain synaptosomes and synaptic vesicles in vitro, *n* = 4–6.

Methiopropamine inhibited the plasmalemmal uptake of DA and NE in rat brain synaptosomes in the submicromolar range (Figure 4a,b), with IC_50_ values of 0.74 ± 0.09 and 0.47 ± 0.06 µM, respectively (Table 1). Methamphetamine was an even more potent inhibitor of the plasmalemmal DA and NE uptake, displaying IC_50_ values of 0.14 ± 0.01 and 0.08 ± 0.00 µM, respectively (Figure 4a,b and Table 1; *p* < 0.01). Both drugs were less potent inhibitors of serotonin (5-HT) uptake with IC_50_ values of 25.14 ± 2.91 µM for methiopropamine and 4.90 ± 0.39 µM for methamphetamine (Figure 4c and Table 1). The vesicular uptake of DA, NE and 5-HT was also inhibited by methiopropamine and methamphetamine (Figure 4d–f), but only at much higher drug concentrations (IC_50_ for methiopropamine: 34–49 µM; IC_50_ for methamphetamine: 11–28 µM; Table 1).

### 2.4. Drug-Induced Changes in Neurotransmitter Concentrations

Brain concentrations of DA, the DA metabolites 3-methoxytyramine (3-MT) and 3,4-dihydroxyphenylacetic acid (DOPAC), 5-HT and the 5-HT metabolite 5-hydroxyindoleacetic acid (5-HIAA) after methiopropamine or methamphetamine exposure were compared with brain neurotransmitter concentrations in control animals injected with saline (Table 2). In crude homogenates of the dorsal striatum (DS), reductions in the DOPAC concentration were seen after administration of 12.5 mg/kg methiopropamine (38% of control, *p* < 0.001), 12.5 mg/kg methamphetamine (29% of control, *p* < 0.001) and 3.75 mg/kg methamphetamine (53% of control, *p* < 0.01). The concentrations of 5-HIAA were also significantly decreased in the DS (65–67% of control, *p* < 0.05) after methiopropamine or methamphetamine administration. A tendency for increased 3-MT concentrations was observed in the DS after injection of 12.5 mg/kg methiopropamine (158% of control, *p* = 0.058) or 12.5 mg/kg methamphetamine (153% of control, *p* = 0.073). In the nucleus accumbens (NAc), the 3-MT concentration was significantly increased after injection of 3.75 mg/kg methamphetamine (369% of control, *p* < 0.05), while all other measured brain concentrations did not differ significantly from those measured in saline-injected animals.

**Figure 5:**
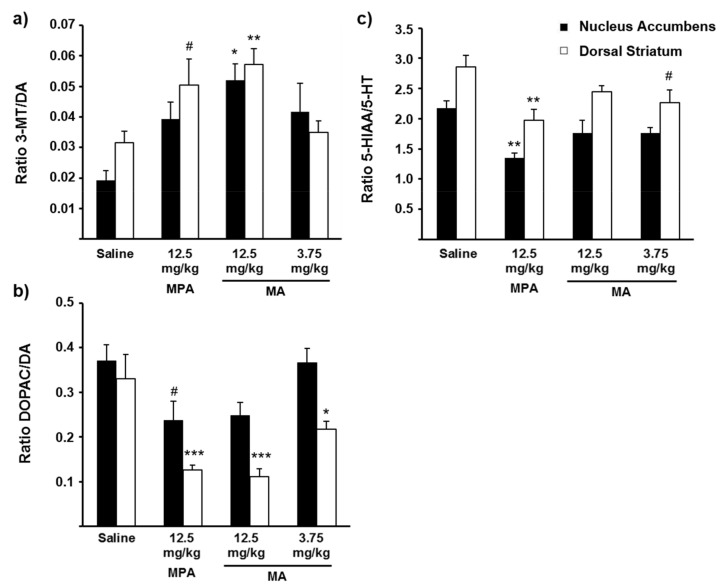
Ratios of (**a**) 3-MT/DA, (**b**) DOPAC/DA and (**c**) 5-HIAA/5-HT in nucleus accumbens and dorsal striatum 20 min after i.p. injection of saline (0.9%), methiopropamine (MPA; 12.5 mg/kg) or methamphetamine (MA; 3.75 or 12.5 mg/kg), *n* = 5–6; * *p* ≤ 0.05; ** *p* ≤ 0.01; *** *p* ≤ 0.001; # *p* = 0.065–0.068 compared with the saline group (Dunnett’s post hoc test). 3-MT, 3-methoxytyramine; 5-HIAA, 5-hydroxyindoleacetic acid; 5-HT, serotonin; DA, dopamine; DOPAC, 3,4-dihydroxyphenylacetic acid.

Figure 5 depicts the ratios of the DA metabolites 3-MT and DOPAC vs. DA, and the 5-HT metabolite 5-HIAA vs. 5-HT in the NAc and DS, measured 20 min after injection of saline, methiopropamine or methamphetamine. The 3-MT/DA ratio was significantly increased after administration of 12.5 mg/kg methamphetamine (DS: 182% of control, *p* < 0.01; NAc: 271% of control, *p* < 0.05), while a tendency to increased 3-MT/DA ratio was seen in the DS after injection of 12.5 mg/kg methiopropamine (160% of control, *p* = 0.065). In comparison, the DOPAC/DA ratio was significantly decreased in the DS after administration of 12.5 mg/kg methiopropamine or methamphetamine (34–38% of control, *p* ≤ 0.001) and 3.75 mg/kg methamphetamine (66% of control, *p* = 0.05), whereas a tendency to decreased ratio was seen in NAc after administration of 12.5 mg/kg methiopropamine (64% of control, *p* = 0.061). Decreased 5-HIAA/5-HT ratios were seen in both the DS (69% of control, *p* < 0.01) and NAc (62% of control, *p* < 0.01) in animals injected with 12.5 mg/kg methiopropamine compared to animals given saline. A tendency for reduced 5-HIAA/5-HT ratio was also seen in the DS after administration of 3.75 mg/kg methamphetamine (79% of control, *p* = 0.068).

## 3. Discussion

Structural modification of classic drugs of abuse is a commonly employed strategy to create NPSs, which circumvents existing drug control legislation. Methiopropamine is one such example where a new recreational drug is created by replacing the benzene ring of methamphetamine with a thiophene ring [9] (Figure 1). The rapid increases in the number and diversity of NPSs on the global drug market remain a major challenge for forensic and clinical laboratories because of the lack of analytical methods and scant information about their pharmacology in humans and laboratory animals. In the present study, we characterized the pharmacokinetics and neuropharmacological effects of methiopropamine in mice, using the structural analog methamphetamine as a reference comparator drug.

The pharmacokinetics of methiopropamine closely resembled the pharmacokinetic profile of methamphetamine, with similar concentration vs. time curves and brain:blood drug ratios. We found that methiopropamine was rapidly absorbed into the blood and brain after i.p. injection in mice, reaching a maximum brain concentration within 10 min.

The elimination half-life was approximately 30 min, which is in the same range as previously reported for methamphetamine [15]. Based on human toxicity reports [9,10,14], and a previous metabolism study suggesting that methiopropamine is metabolized only to a minor extent [7], nor-methiopropamine was the only metabolite included in this study. We found that the nor-metabolite was present at a maximum concentration 24× and 15× lower than the concentration of methiopropamine in the blood and brain, respectively. A recent study examining the urine excretion profile of methiopropamine after administration in mice reported the presence of nor-methiopropamine, oxo-methiopropamine and two hydroxylated metabolites in addition to the unchanged drug [16]. However, only methiopropamine and nor-methiopropamine were detected for a longer time period after exposure, and the authors concluded that these two analytes had the highest diagnostic value in toxicological analysis. It should also be noted that metabolite profiles in urine do not always match those measured in the blood, brain or other tissues.

Amphetamine and its derivatives are well-known inducers of locomotor activity in rodents [17,18,19,20]. Our study shows that high doses of methiopropamine are needed to induce locomotor activity in C57BL/6 mice, with a maximal locomotor effect seen after administration of 12.5 mg/kg methiopropamine, as compared with 3.75 mg/kg for methamphetamine. Importantly, our data show that methiopropamine is more efficacious than methamphetamine as a locomotor stimulant because the former induces a greater extent of overall locomotion. Methiopropamine-induced locomotor stimulation was previously studied in Sprague-Dawley rats [21] and in CD-1 mice [22,23] with a significant increase in locomotor activity seen after i.p. injection of 5 and 10 mg/kg, respectively. At higher methiopropamine doses in mice (30 mg/kg; i.p.), stereotypic behavior, such as an increased number of freezing episodes, rotational behavior and intense repetitive movements, was observed [22]. The observed differences in drug-induced locomotor stimulation in our study compared to the study by De-Giorgio et al. [22] could be due to strain differences between the inbred C57BL/6 and outbred CD-1 mice. In comparison to the gradual/step-wise dose-response increase in locomotor activity seen for methamphetamine, it is worth mentioning the steep increase in locomotor activity observed after a modest increase in the methiopropamine dose from 10 to 12.5 mg/kg. This steep dose-response profile suggests that a small increase in dosage may give unexpectedly strong effects and heighten the risk of unintended overdose in NPS users; however, the clinical significance of this steep dose-response curve in rodents remains to be determined.

Psychostimulants differ in their relative affinity for DA, NE and 5-HT transporters [24], and knowledge of the mechanisms of action of classic psychostimulants can be used to predict differences in the behavioral profiles of novel drugs [25]. As an example, drugs that are selective for the DA transporter are known to be powerful locomotor stimulants, whereas drugs selective for the 5-HT transporter are not [26,27]. In this study, we showed that methiopropamine is a potent inhibitor of the presynaptic NE (IC_50_: 0.45 µM) and DA uptake (IC_50_: 0.66 µM) in isolated rat brain synaptosomes. In contrast, a 30–50× higher methiopropamine concentration was needed to inhibit the plasmalemmal 5-HT uptake to the same extent. This is consistent with a study by Iversen et al. [6] where methiopropamine was shown to act as a potent inhibitor of DA and NE transport activity in HEK293 cells expressing human neurotransmitter transporters, while the 5-HT transport was less affected. When comparing the two substances, the selectivity for the DA, NE and 5-HT transporters was almost identical, with methiopropamine displaying a potency approximately 5× weaker compared with methamphetamine. Nearly identical DAT/SERT ratios of methiopropamine and methamphetamine indicate similar abuse liability for the two drugs [28,29]. Our studies of the effects on the vesicular transporter, VMAT2, revealed an inhibitory effect of methiopropamine and methamphetamine in the micromolar range (IC_50_: ~10–50 µM) with the lowest values found for the vesicular DA uptake (IC_50_: 33.8 µM for methiopropamine and 10.9 µM for methamphetamine). This correlates well with the reported IC_50_ of 9.1 µM for methamphetamine on vesicular DA uptake [30]. Mice exposed to 12.5 mg/kg methiopropamine displayed blood and brain C_max_ values of 3.7 µg/mL and 14.2 µg/g, which equal 24 µM and 91 nmol/g (~91 µM), respectively, suggesting that an inhibitory effect of methiopropamine on vesicular uptake could be relevant after high-dose intake. As a comparison, recreational users driving under the influence of drugs displayed a mean methiopropamine concentration of 0.018 µg/mL (0.12 µM) in the blood [5], whereas a fatal intoxication case reported 38 µg/mL (245 µM) [14]. Camuto et al. [16] estimated a dose of 10 mg/kg in mice to be equivalent to a normal-to-high recreational human dose (~48 mg).

Drug-induced increases in monoamine transmission can be assessed directly by measuring the monoamine levels in brain extracellular fluid, using in vivo microdialysis methods. In the present study, we employed an alternative, albeit less precise, approach by measuring the concentrations of monoamines and their main metabolites in post-mortem tissue from brain areas important to locomotor activation and drug reward. Since the DA metabolite 3-MT can only be formed extracellularly, increases in 3-MT or the 3-MT/DA ratio are thought to be an index of increased DA release from neurons [31,32,33], whereas reduced DOPAC levels or DOPAC/DA ratios reflect reduced intraneuronal DA metabolism [34,35]. Previous studies in rats reported that amphetamine-like stimulants reduce striatal DOPAC concentrations and increase 3-MT concentrations [33,36,37,38]. In animals exposed to the highest dose of methamphetamine (12.5 mg/kg), we observed significantly increased 3-MT/DA ratios in both the DS and NAc, as well as a reduced DOPAC/DA ratio in the DS. Similar findings were seen for methiopropamine; however, the changes in the 3-MT/DA ratio did not reach significance (*p* = 0.065 in the DS). In addition to the effects on the DA system, animals treated with methiopropamine also showed a decreased 5-HIAA/5-HT ratio in DS and NAc, while reduced 5-HIAA levels were induced by both drugs in the DS. The observed changes in the ratio of neurotransmitters vs. neurotransmitter metabolites in brain tissue homogenates are consistent with drug-induced neurotransmitter release (i.e., transporter-mediated neurotransmitter release) and/or inhibited uptake of monoamines into the presynaptic terminal [27]. An additional explanation for the decreases in DOPAC and 5-HIAA produced by both drugs is inhibition of the monoamine metabolizing enzyme monoamine oxidase (MAO) [39,40,41], as previously reported for amphetamine-type drugs [42], including nor-methiopropamine [43]. The MAO inhibitory potential of methiopropamine and other amphetamine-type NPSs is an interesting subject for further studies.

The similarities in mechanisms of action for methiopropamine and methamphetamine observed in this study imply similarities in toxicity as well. Methamphetamine is known to induce neurotoxicity via mitochondrial dysfunction and enhanced oxidative stress (reviewed in [44,45,46]). A recent study examined the neurotoxic potential of methiopropamine in mice and found that repeated methiopropamine exposure (4 × 10–20 mg/kg with 2 h intervals) caused dopaminergic neurotoxicity mediated by oxidative stress, microglial activation and pro-apoptosis [47]. The fact that both methamphetamine and methiopropamine act as submicromolar inhibitors of the NE transporter also suggests common sympathomimetic effects on the cardiovascular system. In a fatal case of methiopropamine intoxication, cardiac arrhythmia followed by cardiovascular collapse was suggested to be the direct cause of death [14]. Sudden deaths were also reported after acute (10 mg/kg; 26–43% deaths) and chronic (10 mg/kg/day for 30 days; 70% deaths) methiopropamine exposure in CD-1 mice [22,48]. In mice, histological analysis revealed myocardial damage consistent with repeated episodes of ischemia [48]. We did not observe any sudden deaths in the C57BL/6 mice exposed to doses up to 20 mg/kg methiopropamine. De-Giorgio et al. [22] reported reduced oxygen saturation in CD-1 mice exposed to methiopropamine. CD-1 mice have previously been shown to be less tolerant to hypoxia than C57BL/6 mice [49,50], and this could possibly contribute to the observed differences in fatality in the two mouse strains.

The present study provides novel information on the pharmacokinetics, mechanism of action and drug potency of methiopropamine, using methamphetamine as a reference comparator drug. The pharmacokinetic study shows that methiopropamine is rapidly distributed to the blood and brain after injection in mice, with a pharmacokinetic profile similar to methamphetamine. Methiopropamine has a selectivity profile at DA, NE and 5-HT transporters that is almost identical to methamphetamine and exerts its effects by inhibiting NE and DA uptake at submicromolar concentrations. A limitation of the present study is that the behavioral experiments only addressed locomotor activity, while future studies should examine potential abuse liability. Furthermore, the neurochemical experiments examined monoamines in post-mortem brain tissue, whereas measuring monoamine levels in brain extracellular fluid by in vivo microdialysis would provide more precise information.

In summary, methiopropamine closely resembles methamphetamine with regard to its pharmacokinetics, pharmacodynamic effects and mechanism of action, with a potency approximately five times lower than methamphetamine. The steep increase in locomotor activity seen after a modest increase in the methiopropamine dose from 10 to 12.5 mg/kg suggests that small increases in dosage may give unexpectedly strong effects and increase the risk of unintended overdose in NPS users.

## 4. Materials and Methods

### 4.1. Drugs and Chemicals

Drugs: Methiopropamine hydrochloride (HCl) (mol. wt. 191.7) was purchased from Cayman Chemicals (Middlesex, UK) and (+)methamphetamine HCl (mol. wt. 185.7) was purchased from Chiron AS (Trondheim, Norway). Chemicals: 5-HT HCl, L-NE HCl, 5-HIAA HCl, 3-MT HCl, ephedrine-D_3_, sodium fluoride, methanol (LC-MS Chromasolv^®^), ammonium formate, benztropine mesylate, reboxetine mesylate hydrate, fluoxetine HCl and adenosine 5′-triphosphate disodium salt hydrate were purchased from Sigma-Aldrich (Oslo, Norway). DA HCl and DOPAC were purchased from Fluka (Buchs, Switzerland). DA-^13^C_6_ HCl, 5-HT-D_4_ HCl, 5-HIAA-D_5_, methamphetamine-^13^C_6_ and nor-methiopropamine were purchased from Chiron AS. Di-sodiumtetraborate-10-hydrate and ethyl acetate were purchased from Chemi-Teknik AS (Oslo, Norway). Acetic acid and formic acid were purchased from VWR International AS (Oslo, Norway). Sodium hydroxide and n-heptane were purchased from Merck Millipore (Oslo, Norway). Dihydroxyphenylethylamine 3,4-[ring-2,5,6-^3^H]-(DA) ([^3^H]DA), NE HCl DL-[7-^3^H(N)] ([^3^H]NE), 5-HT [^3^H(G)] ([^3^H]5-HT) and UltimaGold^TM^ liquid scintillation cocktail were purchased form from PerkinElmer Inc. (Oslo, Norway). HEPES was purchased from Thermo Fischer Scientific (Oslo, Norway) and sodium heparin was purchased from Leo Pharma (Oslo, Norway).

### 4.2. Animals

Male C57BL/6J mice (7–8 weeks old, 20–25 g; Taconic, Ejby, Denmark) and Sprague-Dawley rats (6–8 weeks old, 150–225 g, Janvier Labs, Saint-Berthevin, France) were housed with 4–8 mice/cage and 2 rats/cage in the animal facility at the Norwegian Institute of Public Health (Oslo, Norway). Animals were given free access to water and commercial food pellets for rodents. Temperature, humidity and lighting were regulated (22 ± 1 °C, 50 ± 10% humidity, light period 7:00 AM–7:00 PM). The animals were acclimatized for at least five days prior to the experiments. The animal experiments were approved by the Norwegian Animal Research Authority (protocol code: 7462; Norwegian Food Safety Authority, Oslo, Norway) and performed in accordance with the laws and regulations controlling experiments on animals in Norway.

### 4.3. Locomotor Activity Studies in Mice

Locomotor activity was assessed using the Versamax optical animal activity monitoring system (AccuScan Instruments Inc., Columbus, OH), as described in detail by Andersen et al. [51]. Briefly, mice were placed individually in an activity chamber for 60 min of habituation. After habituation, each mouse was given an intraperitoneal (i.p.) injection (10 mL/kg) of vehicle (0.9% saline), methiopropamine (5–20 mg/kg) or methamphetamine (1–5 mg/kg). Immediately thereafter, each mouse was returned to its respective activity chamber and locomotor activity recorded for 240 min. Horizontal movement was detected by infrared beams and corresponding photodetectors. Locomotor activity was expressed as the distance (cm) travelled per 5 min interval, maximum activity per 5 min interval (E_max_) or as the total run distance during the 240 min session.

### 4.4. Pharmacokinetic Studies of Methiopropamine and Methamphetamine in Mice

#### 4.4.1. Sampling of Blood and Brain Tissue

Mice were given a single i.p. injection of methiopropamine (12.5 mg/kg) or methamphetamine (3.75 mg/kg) and anaesthetized with isoflurane prior to blood and brain tissue sampling. To assess the level of anesthesia, the pedal reflex was tested by a firm toe pinch. Blood sampling by heart puncture was performed at different time points after drug injection (5–240 min) using a syringe prefilled with heparin (final concentration 12–13 IU/mL). Blood samples (50 or 100 µL) were transferred to tubes containing 100 µL ice-cold 5 mM ammonium formate buffer, pH 3.1, with sodium fluoride (final conc. 4 mg/mL), and were immediately frozen in liquid nitrogen. After cervical dislocation, the cerebrum was quickly removed, homogenized (4 mL per g tissue) in ice-cold 5 mM ammonium formate buffer, pH 3.1, containing sodium fluoride (4 mg/mL), aliquoted (50 or 100 µL) and frozen in liquid nitrogen. Blood and brain samples taken 240 min after drug injection were from animals included in the locomotor activity study. The samples were stored at −80 °C until sample preparation and determination of drug concentrations.

#### 4.4.2. Sample Preparation and UPLC-MS/MS Analysis

Stock solutions of analytes and internal standards were prepared in methanol and stored at −20 °C. Working solutions for seven calibrators and four quality control (QC) samples were prepared in 5 mM ammonium formate buffer. Calibrators (0.10–20 µM) and QC samples (0.10–15 µM) were prepared independently by fortifying human whole blood or rat brain tissue homogenate with working solutions. Samples and solutions were kept on ice throughout the experiment. An internal standard (50 µL, 5 µM ephedrine-d_3_ and methamphetamine-^13^C_6_ in 5 mM ammonium formate buffer, pH 3.1) was added to all samples followed by immediate agitation on a multitube vortexer. A borate buffer (100 µL, pH 11) and ethyl acetate/heptane mixture (1200 µL, 4:1, *v*/*v*) were added, the samples shaken for 10 min and then centrifuged for 10 min at 3900× *g* at 4 °C. The organic layer was transferred to glass tubes and 0.1% HNO_3_ (30 µL in methanol) was added before drying under a stream of nitrogen at 40 °C. The samples were reconstituted (400 µL, 10 mM ammonium formate buffer, pH 3.1/methanol (90:10, *v*/*v*)) and thoroughly shaken prior to transfer to autosampler vials.

Chromatographic separations were performed using an Acquity™ ultra-performance liquid chromatography (UPLC) system (Waters, Milford, MA, USA), applying an Acquity™ UPLC HSS T3 column (2.1 × 100 mm, 1.8 µm; Waters) with an Acquity™ UPLC HSS T3 VanGuard pre-column (2.1 × 5 mm, 1.8 µm; Waters). The mobile phase consisted of 10 mM ammonium formate buffer, pH 3.1 (A) and methanol (B). The separation was carried out using a 7-min gradient with the following profile: 0–4.0 minutes; 2.5–22.5% B, 4.0–4.1 min; 22.5–100% B, 4.1–6.0 min; 100% B, 6.0–6.01 min; 100–2.5% B, and 6.01–7.0 min; 2.5% B. The flow rate was 0.5 mL/min, the column temperature 65 °C and the injection volume 0.5 µL.

A Waters Quattro Premier XE tandem mass spectrometer (MS/MS), or a Xevo TQ triple quadrupole mass spectrometer, equipped with a Z-spray electrospray interface, was used for the analyses. Positive ionization was performed in the multiple reaction monitoring mode, with two transitions for all analytes (methiopropamine: 156.2 > 124.8/96.7; nor-methiopropamine: 142.1 > 125.0/97.0; methamphetamine: 150.1 > 91.1/119.1), and one transition for methamphetamine-^13^C_6_ (156.2 > 125.1) and ephedrine-d_3_ (169.1 > 117.0). The retention times for methiopropamine, nor-methiopropamine and ephedrine-d_3_ were 3.05, 2.82 and 2.97 min, respectively, while methamphetamine and methamphetamine-^13^C_6_ both eluted at 3.86 min. Quantification was performed using MassLynx 4.2 software (Waters). Calibration curves were constructed by plotting the calibrator concentration against the analyte/internal standard peak height ratio. The internal standard used for methiopropamine and nor-methiopropamine was ephedrine-d_3_ and the internal standard used for methamphetamine was methamphetamine-^13^C_6_. The calibration curves were linear, and the QC sample results were satisfactory (≤±20% deviation from the nominal values).

### 4.5. Determination of Neurotransmitters and Neurotransmitter Metabolite Concentrations

#### 4.5.1. Sampling of Mouse Brain Tissue

Mice were given a single i.p. injection of methiopropamine (12.5 mg/kg), methamphetamine (3.75 or 12.5 mg/kg) or saline, anesthetized (as described in Section 4.4.1) and sacrificed by cervical dislocation 20 min later. The brain was removed, and the cerebrum placed in a brain matrix (AgnTho’s AB, Lidingö, Sweden) to cut a 2 mm coronal brain slice. Samples from the NAc and DS were collected from both hemispheres of the slice by micropunches (1 mm, Fine Science Tools, Heidelberg, Germany). The brain samples were immediately frozen in liquid nitrogen and stored at −80 °C until sample preparation and determination of neurotransmitter and neurotransmitter metabolite concentrations within the same day.

#### 4.5.2. Sample Preparation and UPLC-MS/MS Analysis

Stock standard solutions of DA, 5-HT, 3-MT, DOPAC and 5-HIAA were prepared in 25 mM formic acid, stored at 4 °C and diluted to nine calibrators (1.0–80,000 nM) and three QC samples (150, 300 and 3000 nM). The brain samples were thawed and immediately sonicated in 0.1 M formic acid (15–35 mL/mg tissue) using an ultrasonic cell disrupter (2 × 1 s bursts). Brain homogenate (50 µL) and internal standard (25 µL, 3 µM DA-^13^C_6_, 5-HT-d_4_ and 5-HIAA-d_5_ in 25 mM formic acid) were added to plastic tubes and vortexed for 20 s. The samples were centrifuged for 10 min at 14,500× *g* and 4 °C, and the supernatant transferred to amber colored autosampler vials. For calibrators and QC samples, 50 µL was added to 25 µL internal standard in amber colored vials. The concentrations of neurotransmitters and neurotransmitter metabolites in NAc and DS were determined by an UPLC-MS/MS method described by Bergh et al. [52]. Detection was performed with a Xevo TQ triple quadrupole mass spectrometer (Waters, Milford, MA, USA). The calibration curves were linear, and the QC sample results were satisfactory (≤±20% deviation from the nominal values).

### 4.6. Neurotransmitter Uptake Studies

#### 4.6.1. Isolation of Synaptosomes and Synaptic Vesicles from Rat Brain

Synaptosomes from rat cerebrum were isolated as described by Bogen et al. [53]. In brief, the rats were decapitated and their brains rapidly removed. The cerebrum was homogenized in ice-cold 0.32 M sucrose (5%, *w*/*v*) using a glass-Teflon homogenizer at 450 r.p.m. The homogenate was centrifuged at 1000× *g* at 2 °C for 10 min to remove nuclei and cellular debris. The supernatant was mixed 1:1 with 1.3 M sucrose to obtain a final concentration of approx. 0.8 M, and centrifuged at 21,000× *g* at 2 °C for 30 min. The supernatant was discarded and the synaptosomal fraction (white layer of the pellet) was gently resuspended in 6 mL ice-cold 0.32 M sucrose. Synaptosomes were used within 2 h after isolation.

Synaptic vesicles from rat cerebrum were isolated as described previously [53]. The cerebrum was homogenized in ice-cold 0.32 M sucrose (5%, *w*/*v*) and centrifuged at 1000× *g* at 2 °C for 10 min. The supernatant was centrifuged at 21,000× *g* at 2 °C for 30 min. The pellet was osmotically shocked by resuspension in 18 mL ice-cold Type 1 water, and centrifuged at 21,000× *g* at 2 °C for 30 min. The supernatant was collected and 2 mL 1 M K-Tartrate in 0.25 M HEPES was added. This mixture was centrifuged at 2 °C at 100,000× *g* for 1 h. The pellet was gently resuspended in 5.6 mL 0.32 M sucrose, aliquoted and snap frozen in liquid nitrogen. The synaptic vesicles were stored at −150 °C until use.

#### 4.6.2. Synaptosomal and Vesicular Uptake of Neurotransmitters

Synaptosomal uptake of neurotransmitters was determined as described by Bogen et al. [53]. Synaptosomes (20 or 40 µL) were pre-incubated for 15 min at 25 °C in Tris-Krebs buffer (10 mM Tris, 140 mM NaCl, 5 mM KCl, 5 mM NaHCO_3_, 1 mM MgCl_2_, 1.2 mM Na_2_HPO_4_, 1.2 mM CaCl_2_, 10 mM glucose) with different concentrations of methiopropamine (0.1–50 µM) or methamphetamine (0.05–50 µM). The uptake was initiated by adding either 0.025 µM [^3^H]DA (0.5 µCi), 0.014 µM [^3^H]NE (0.1 µCi) and 0.06 µM NE, or 0.012 µM [^3^H]5-HT (0.5 µCi) and 0.08 µM 5-HT (final concentrations) in a total volume of 500 µL. After 9 min incubation, the reactions were terminated by dilution with ice-cold wash solution (0.15 M NaCl and 0.05% (*w*/*v*) bovine serum albumin) and rapid filtration through GF/B glass microfiber filters (25 mm, VWR International AS, Oslo, Norway). The filters were washed 4×, dissolved in 4 mL UltimaGold^TM^ liquid scintillation cocktail and counted for the retained radioactivity in a liquid scintillation analyzer (Tri-Carb 2810TR, PerkinElmer Inc., Oslo, Norway). Non-specific uptake tubes were treated similarly, but incubation was performed either in the presence of the DA transporter inhibitor benztropine mesylate (0.01 µM), the NE transporter inhibitor reboxetine mesylate (0.05 µM) or the 5-HT transporter inhibitor fluoxetine (0.05 µM). The specific uptake was defined as the total uptake minus the non-specific uptake.

Synaptic vesicles (12.5 µL) were pre-incubated for 15 min at 30 °C in incubation buffer (6 mM KCl, 6 mM MgSO_4_, 0.19 M sucrose, 15 mM HEPES/KOH, pH 7.4) with different concentrations of methiopropamine (0.1–50 µM) or methamphetamine (0.1–50 µM). The uptake was initiated by adding 1.8 mM ATP and either 0.025 µM [^3^H]DA (0.5 µCi), 0.068 µM [^3^H]NE (0.5 µCi) or 0.012 µM [^3^H]5-HT (0.5 µCi) in a total volume of 330 µL. After incubation for 3 min, the reactions were terminated by dilution with ice-cold wash solution (0.15 M KCl) and rapid filtration through MFTM membrane filters (0.45 µm HA, Merck Millipore). The filters were washed 4×, dissolved in 4 mL UltimaGold™ and counted for retained radioactivity in a liquid scintillation analyzer. Non-specific uptake tubes were treated similarly, but incubation was performed in the presence of 25 µM reserpine. The specific uptake was defined as the total uptake minus the non-specific uptake.

### 4.7. Data and Statistical Analysis

Data are expressed as mean ± SEM, or mean + SEM, unless stated otherwise. Animals were randomly assigned to the different experimental groups. The pharmacokinetic analyses and neurotransmitter uptake experiments were performed in duplicate samples. Elimination half-lives were calculated from the equation t_1/2_ = ln 2/k, where k is the elimination rate constant (k = ln C_1_ − ln C_2_/t_2_ − t_1_) [54]. For calculation of the concentration ratio in brain vs. blood for methiopropamine and methamphetamine, we approximated that the densities of brain homogenate and blood were equivalent (i.e., µg/g ≈ µg/mL). IC_50_ values were calculated by nonlinear regression ([inhibitor] vs. normalized response-variable slope) using Graph Pad Prism version 7.02 for Windows (GraphPad Software, La Jolla, CA, USA). Statistical analysis of drug-induced locomotor activity over time was performed using the General Linear Model for repeated measures, followed by Dunnett’s post hoc test. One-way ANOVA was performed for comparison of total locomotor activity, E_max_, effects on neurotransmitter concentrations and concentrations ratios, with a significant F value followed by Dunnett’s post hoc test. Independent samples *t*-tests were performed for comparison of the IC_50_ values and comparison of the brain:blood ratios for methiopropamine and methamphetamine. *P* values less than 0.05 were considered statistically significant. All statistical tests were performed using SPSS, version 26 (SPSS Inc., Chicago, IL, USA).

## Figures and Tables

**Table 1:** Effects of methiopropamine (MPA) and methamphetamine (MA) on uptake of [^3^H]DA, [^3^H]NE and [^3^H]5-HT in rat brain synaptosomes and synaptic vesicles.

IC_50_ Synaptosomes	[^3^H]DA Uptake via DAT (µM)	[^3^H]NE Uptake via NET (µM)	[^3^H]5-HT Uptake via SERT (µM)
MPA	0.74 ± 0.09 **	0.47 ± 0.06 **	25.14 ± 2.91 ***
MA	0.14 ± 0.01	0.08 ± 0.00	4.90 ± 0.39
**IC_50_** **Synaptic Vesicles**	**[^3^H]DA Uptake via VMAT2 (µM)**	**[^3^H]NE Uptake via VMAT2 (µM)**	**[^3^H]5-HT Uptake via VMAT2 (µM)**
MPA	33.79 ± 12.47	47.00 ± 8.01	48.89 ± 5.02 *
MA	10.92 ± 1.18	23.90 ± 8.55	28.23 ± 3.54

DA, dopamine; DAT, dopamine transporter; IC_50_, half maximal inhibitory concentration; NE, norepinephrine; NET, norepinephrine transporter; SERT, serotonin transporter, VMAT2, vesicular monoamine transporter 2; 5-HT, serotonin. *n* = 4–5; * *p* < 0.05; ** *p* < 0.01; *** *p* < 0.001 vs. methamphetamine (*t*-test, independent samples).

**Table 2:** Concentrations (nmol/g) of DA, the DA metabolites 3-MT and DOPAC, 5-HT and the 5-HT metabolite 5-HIAA 20 min after i.p. injection of methiopropamine (MPA) or methamphetamine (MA).

	DA	3-MT	DOPAC	5-HT	5-HIAA
Dorsal Striatum					
Saline	128.9 ± 15.7	3.9 ± 0.3	41.2 ± 6.0	2.4 ± 0.3	6.7 ± 0.7
12.5 mg/kg MPA	126.6 ± 15.1	6.1 ± 0.8 #	15.6 ± 1.6 ***	2.3 ± 0.2	4.5 ± 0.5 *
12.5 mg/kg MA	109.6 ± 19.4	5.9 ± 0.6 #	12.1 ± 2.6 ***	1.8 ± 0.2	4.3 ± 0.6 *
3.75 mg/kg MA	107.4 ± 21.1	3.6 ± 0.7	22.0 ± 3.2 **	2.0 ± 0.3	4.4 ± 0.6 *
**Nucleus Accumbens**					
Saline	62.4 ± 7.9	1.0 ± 0.2	30.3 ± 10.0	5.0 ± 0.8	10.6 ± 1.5
12.5 mg/kg MPA	62.5 ± 6.9	2.5 ± 0.5	14.9 ± 1.9	5.2 ± 0.9	6.8 ± 1.0
12.5 mg/kg MA	54.6 ± 10.4	2.9 ± 0.4	12.5 ± 2.7 #	5.6 ± 1.4	8.4 ± 1.9
3.75 mg/kg MA	82.2 ± 10.5	3.5 ± 1.0 *	28.8 ± 2.6	6.1 ± 0.8	10.6 ± 1.4

*n* = 5–6; * *p* ≤ 0.05; ** *p* ≤ 0.01; *** *p* ≤ 0.001; # *p* = 0.056–0.073 vs. saline (Dunnett’s post hoc test).

## Abbreviations

DA, dopamine; DAT, dopamine transporter; DOPAC, 3,4-dihydroxyphenylacetic acid; DS, dorsal striatum; IC_50_, half maximal inhibitory concentration; MA, methamphetamine; MPA, methiopropamine; NAc, nucleus accumbens; nor-MPA, nor-methiopropamine; NE, norepinephrine; NET, norepinephrine transporter; QC, quality control; SERT, serotonin transporter; VMAT2, vesicular monoamine transporter 2; 5-HIAA, 5-hydroxyindoleacetic acid; 5-HT, serotonin; 3-MT, 3-methoxytyramine; UPLC-MS/MS, ultra-performance liquid chromatography tandem mass spectrometry.
